# Bridge Damage Detection Approach Using a Roving Camera Technique

**DOI:** 10.3390/s21041246

**Published:** 2021-02-10

**Authors:** Darragh Lydon, Myra Lydon, Rolands Kromanis, Chuan-Zhi Dong, Necati Catbas, Su Taylor

**Affiliations:** 1 School of Natural and Built Environment, Queen’s University Belfast, Belfast BT7 1NN, UK; d.lydon@qub.ac.uk (D.L.); s.e.taylor@qub.ac.uk (S.T.); 2 Department of Civil Engineering, University of Twente, Drienerlolaan 5, 7522 NB Enschede, The Netherlands; r.kromanis@utwente.nl; 3 Department of Civil, Environmental, and Construction Engineering, University of Central Florida, Orlando, FL 32816, USA; ceczdong@knights.ucf.edu (C.-Z.D.); catbas@ucf.edu (N.C.)

**Keywords:** computer vision, damage detection, structural health monitoring, sensor roving

## Abstract

Increasing extreme climate events, intensifying traffic patterns and long-term underinvestment have led to the escalated deterioration of bridges within our road and rail transport networks. Structural Health Monitoring (SHM) systems provide a means of objectively capturing and quantifying deterioration under operational conditions. Computer vision technology has gained considerable attention in the field of SHM due to its ability to obtain displacement data using non-contact methods at long distances. Additionally, it provides a low cost, rapid instrumentation solution with low interference to the normal operation of structures. However, even in the case of a medium span bridge, the need for many cameras to capture the global response can be cost-prohibitive. This research proposes a roving camera technique to capture a complete derivation of the response of a laboratory model bridge under live loading, in order to identify bridge damage. Displacement is identified as a suitable damage indicator, and two methods are used to assess the magnitude of the change in global displacement under changing boundary conditions in the laboratory bridge model. From this study, it is established that either approach could detect damage in the simulation model, providing an SHM solution that negates the requirement for complex sensor installations.

## 1. Introduction

The progressive deterioration of civil infrastructure is now of paramount concern to asset owners and users alike. Particularly in relation to bridge structures across the road and rail networks, damage accumulated due to overloading, extreme weather conditions and general fatigue has put the overall network at risk. Structural damage results in a change in the geometric or material properties of bridges, which manifests in the forms of stiffness, mass, damping, and the boundary condition and stability changes of structures. Traditional bridge inspections are sensitive to human error and bias, and can often result in overly-conservative assumptions of reduced load-carrying capacity [1]. Structural Health Monitoring (SHM) systems provide a means of objectively capturing and quantifying this change under operational conditions. The application of such systems has significant cost-saving potential across the lifespan of bridge structures, and can ensure the safe operation of our road and rail transport networks. With over one million bridges across Europe, the task of assessing each structure often surpasses the available resources. This shortfall dramatically reduces the resilience of transport networks and increases vulnerabilities, particularly considering that 35% of Europe’s rail bridges are over 100 years old [2]. Understanding the true capacity of ageing civil infrastructure is now more critical than ever, as an increasing number of failure events in clusters of bridges have occurred, such as those witnessed in Cumbria in 2007, Northern Ireland in 2017, and Yorkshire in 2019 [3,4,5]. This risk extends beyond purely economic considerations, as in 2009 fatalities were narrowly avoided when the Malahide railway bridge collapsed north of Dublin city just seconds after a passenger train crossed the structure [6]. Italy was less fortunate in 2018, when at total of 43 people lost their lives due to the Morandi Bridge collapse in Genoa [7]. Since 2018, Italy has witnessed two additional major bridge failures; it is thought that the COVID-19 lockdown conditions were a key factor in avoiding fatalities related to the most recent event, the collapse of Albiano bridge, which connects the Italian cities of Genoa and Florence, in April 2020 [8]. In response to this, during the last two decades, a significant amount of research has been dedicated to the development and enhancement of SHM systems for bridge monitoring. However, the challenge of accurately detecting and quantifying damage in civil infrastructure still exists globally, and few systems have been deployed and verified on real bridges.

## 2. Literature Review

### 2.1. Damage Detection Methods

SHM systems are used to collect bridge response data, which can then be analysed to detect damage indicators and provide evidence of a change in the condition or heath of the bridge. Typically, a bridge is considered safe if the probability of a load effect is significantly less than the corresponding resistance or capacity. Probabilistic and reliability-based approaches to bridge assessment have been widely used to quantify bridge safety [9,10,11,12,13]. In reality, the problem is that the damage indicator tends to be incompatible with the information used to assess the safety and maintenance [14]. Structural damage detection is typically carried out using vibration-based analysis; the recent developments and established methods in this area have been summarised in a review paper [15]. The most commonly utilized technique in recent decades is modal-based damage detection [16,17,18,19,20,21]. Modal analysis is a widely accepted method of bridge analysis, and it has had very successful commercial application worldwide. Traditionally, changes in natural frequencies, mode shapes, mode curvatures or damping ratios have been used as a damage indicator. A comparative study of the modal-based damage detection techniques has found that the method is very sensitive to noise contamination, and can only successfully detect severe levels of damage [22]. Environmental effects have been found to have a significant effect on modal-based methods [23]. Such approaches have been found to perform well in controlled laboratory or theoretical conditions, but are extremely difficult to implement in the field. In many cases, temperature variation can have a greater effect on the dynamic behaviour of the bridge than the presence of damage. This can result in false damage identification, or—in some cases—can obscure the detection of real damage [24,25]. The effect can be reduced through data normalisation and statistical training methods, such as principal component analysis [26].

Artificial intelligence (AI) methods have been found to improve the robustness of this technique, but the drawbacks of modal based analysis are still prevalent and have led to the investigation of alternative methods of damage detection. AI methods—such as adaptive neuro-fuzzy interference systems (ANFIS)—have been found to provide a high degree of accuracy for structural response, and—when coupled with interval modelling—can effectively extract damage indicators [27]. The method was found to identify damage within 0.03 s of its occurrence in a series of finite element models but, as with many damage detection methods, it has not been applied in the field. A method which was successfully trialled in experimental laboratory conditions was the use of 1D Convolutional Neural Networks (CNNs) for real-time damage detection [28]. Large-scale experiments were carried out on a grandstand simulator at Qatar University; in cases of a single damage location, the CNN correctly identified the damage location in all 18 test cases. For field applications, a low-cost monitoring system is made feasible by the simple structure of the CNN, and its inexpensive computational demands lend it suitability for field SHM applications. A downfall of the system is that the data for a damaged structure is required to train the CNN, which infers it to be currently unsuitable for field applications, as it is difficult to obtain training data for bridge damage scenarios. Alternative AI methods include the use of autoencoder-based frameworks in deep neural networks, which can provide a solution for damage detection in non-linear cases [29]. The framework was tested in both numerical and experimental conditions utilizing the pattern recognition of model information; the output stiffness reduction parameters provided the damage indicator using a regression model. The technique performed well both in numerical and experimental conditions, which allowed for environmental conditions which may be encountered on site. Other vibration-based analysis includes the interpolation damage detection method (IDDM); in this case, the damage index is defined in terms of deformed shapes obtained from the frequency response functions [30]. The main drawback of this method is the assumptions that are made if data for the structure in its undamaged state is not available, making it unsuitable for some field applications. 

More recently, a symbiotic data-driven approach has been developed based on clustering analysis, which reduces the raw vibration data into representative sets with the capabilities for real time monitoring [26]. This method is particularly useful for the application of SHM to ageing structures, as it does not require baseline data. In this study, the dynamic cloud clustering algorithm was used; this assesses large data sets from multisensory systems. A cluster validity index then evaluates the quantitative descriptive measure of cluster compactness, thereby reducing the possibility of a false positive outlier. Cluster analysis can be seen as an alternative approach because it does not require a prior baseline to perform feature discrimination, which is useful for the health assessment of aged structures, or for post-accident/post-retrofitting situations. Recent studies in damage detection have provided real-time solutions using recursive principal component analysis in conjunction with time varying auto-regressive modeling to successfully detect instantaneous structural damage [31,32,33]. As a recursive model updating approach, the Kalman filter (EKF) has been used in combination with various regularization methods to identify structural parameters and their changes in both vibration- and vision-based data. Given its favourable performance in arithmetic robustness, identification accuracy, and fast convergence, EKF has been extensively applied to vibration-based measurement, with effective outcomes [34,35,36]. When applied to vision-based measurements, it was found to perform well in small-scale testing, but was less suitable for large-scale structures subject to ambient vibrations [37,38].

Overall, there is no absolute consensus on which vibration-based technique is most suitable for bridge SHM, as many of the documented methods have challenges in their implementation in the field. The requirement of a denser array of sensors for accurate modal curvatures at higher modes can often be prohibitive in field applications due to access issues of power requirements [15]. AI has made the processing and storage of vibration data significantly more cost efficient. However, limitations still exist in the training of such systems for accurate damage identification. This paper introduces a displacement monitoring approach, providing a low cost and computationally-efficient method which can be implemented for bridge damage detection using computer vision-based SHM.

Displacement measurements provide a valuable insight into the structural condition and service behaviour of structures under live loading. Displacement has been used as a metric for bridge condition rating in numerous studies outlined in the following section. The analysis of the monitored displacement values over time can provide an insight into possible excessive loading or changes to structural behaviour, since displacement can be directly linked to structural stiffness and external loading. In a long term analysis (multiple years), the displacement responses can be used to create a pattern of structural response to temperature or vehicle loading; if the measured responses display extreme variance from this pattern, it could be surmised that there has been a change to the structural properties of the monitoring subject. In [39], a displacement curve was used to detect and localise the damage of a cantilever beam structure. The excitation of a cantilever beam was also used in a study in [40], in which integrated accelerations were used to calculate a displacement response used to inform a damage detection model. The integration of acceleration data to produce displacement measurements is a common procedure; however, it is prone to measurement noise or bias, resulting in errors in the calculated displacement values [41]. In [42], Zhang et al. used the displacement caused by a vehicle passing over a bridge structure as a modelling scenario for simulated damage detection. This type of experiment, where the changes to the displacement curvature based on repeated passes by a vehicle established the methodology of the laboratory work described in this paper. A test set up by the authors, in which a bridge model is fully instrumented to determine displacement from a passing vehicle, is laid out in [43]. Damage was detected under several scenarios in this study, with localised damage detected at multiple instances of location and severity. This was developed further in [44], in which a series of accelerometer clusters were used to detect damage under a sensor roving technique. The research presented below will further enhance the development of the sensor roving technique with a camera roving technique for damage detection by replacing the cumbersome setup of the accelerometer cluster with a set of time-synchronised cameras for displacement measurement. These studies all demonstrate the use of displacement measurement as a powerful means of assessing bridge condition through performance monitoring.

### 2.2. Computer Vision-Based Displacement Measurement Methods

With the advantages of being long distance, non-contact, and low cost, and having rapid instrumentation and minimal interference to the normal operation of structures, displacement measurements using computer vision technology have gained significant attention in the field of SHM [45]. These studies can be organised into several categories. 

#### 2.2.1. Single Camera Studies on Artificial Targets

One of the first studies carried out in this area was the work on the Humber Bridge using targets in conjunction with a template-matching method performed by [46]. In this research, a camera with a zoom lens was focused on a high-contrast target that was installed at the midspan of the Humber Bridge. Due to limitations of storage capacity, a limited frame rate of 4.17 frames per second (FPS) was used for this testing, which limits the information gathered from live loading, as the peak deflections can be missed at this rate. The results were compared to integrated acceleration values, but only a visual comparison of the results was presented in the paper. While the lack of statistical comparison between the vision-based results and the traditional instrumentation is unfortunate, the results of the work were promising, and were an early step in demonstrating the viability of the use of a camera to study bridge displacement. This experimental style, in which a single camera was pointed at the midspan of a bridge, has been repeated several times in multiple studies with increasing accuracy, as camera technology and image processing systems have developed over time. Dynamic testing at 30FPS was carried out using a template matching system by [47]; the initial testing of their proposed system was carried out in the laboratory and verified by accelerometers. While this verification method is useful for dynamic testing scenarios, it was explained in Section 2.1 that displacement readings from integrated accelerations are unreliable. A dynamic field trial was performed in which LEDs were installed on a bridge structure and tracked under live loading; this test was essentially a proof-of-concept, as no measurement verifications were presented. One of the first studies to show the reliable verification of dynamic displacement loading was the research performed by [48]. This study performed a live-loading scenario on a 40 m span box-girder bridge. The testing involved tracking the movement of a target placed at the midspan while trucks moved across the bridge at speeds varying from 30 to 50 km/h. The displacement measurements were gathered in real time and analysed using a correlation-based system with verification provided by a laser vibrometer, which was shown to be extremely accurate [49]. The correlation between the laser measurement and their computer vision method was not published; furthermore, the error from the measurements was only generally discussed (approximately 10%, compared to the laser). As camera technology has improved in tandem with progressively more accurate means of tracking keypoints through a video, the tracking of natural features on a bridge structure has become increasingly viable.

#### 2.2.2. Single Camera Studies on Natural Bridge Features

An early study that produced verifiable non-contact displacement measurement was the work of [50]. This study used an orientation code-matching algorithm [51] to obtain displacement measurements from natural features on the bridge. Field testing was performed by focusing two synchronised cameras on the midspan of the bridge; camera A was focused on a target pattern attached to the bridge, and camera B was focused on the natural features of the bridge. Previous work by the authors in [52] used the FREAK [53] binary descriptor to monitor the displacement of a stadium structure during a football game, with measurements verified by LVDT. The combination of FREAK and a geometric transformation method for outlier removal resulted in extremely accurate results in comparison to the LVDT measurements. The correlation coefficient (CC) between the reference and vision data was found to be 0.9880 at a monitoring distance of 7 m, with a determination coefficient of 0.9426 for the same monitoring distance. The displacement of a model beam structure was measured using a smartphone camera with accurate results compared to LVDT in the work in [54].

#### 2.2.3. Multiple Camera Studies

Displacement measurement using a single camera is useful, but in order to obtain a full picture of the displacement response to loading, a multiple camera setup is required. The damage detection method proposed in this paper requires the synchronised measurement of the displacement of multiple points along the bridge span. Multiple camera systems generally consist of several cameras synchronised by means of a ‘master–slave’ relationship, in which a central unit controls the timing of any recordings made by the cameras connected to it. This principle was first demonstrated in the area of bridge displacement monitoring by [55]. The authors synchronised the cameras by means of a wireless TCP/IP protocol in which the master unit corrected for any drift in times between the slave units. The synchronisation method was only verified in a single laboratory trial, in which the slave units were placed 10 m away from the control unit, the effects of signal loss from the control unit, and from monitoring at a greater separation distance were not explored. The results of two cameras monitoring a single location appear to agree well, though this cannot be verified, as no numerical data was provided to determine the correlation of the two cameras. A partitioning method for the study of the displacement of a high rise building was developed in [56], in which paired cameras were used in series to monitor progressively higher levels of a tall building. Theoretically, this system would result in a detailed analysis of the building’s response; unfortunately, in this study, the authors did not explain or verify that their multiple cameras were accurately synchronised, so this application cannot be considered a viable multiple camera approach. The research in the previous two studies was continued by [57], in which a wireless TCP/IP time synchronised system was demonstrated, along with the application of this system for the partitioning approach. A disadvantage of this method is the requirements on site: one PC as a master unit, one PC per camera, plus a router and frame grabber. This study also does not demonstrate what would occur if the master unit was removed from the system, which could be an issue if the system is to be left unattended for long-term monitoring. A long range, wireless system featuring millisecond-level synchronisation was developed and validated by the authors in [58]. This system consisted of a master–slave synchronisation method with timelock data transferred via Radio Frequency in order to allow for accurate metadata insertion into all of the recorded frames.

This paper is focused on the testing of this master-slave synchronisation method to provide of a cost-effective SHM solution for damage detection for short- to medium-span bridges using displacement as a damage indicator. Previous studies have shown that the dynamic response has a greater influence for damage detectability under varying temperatures than static bridge response. Therefore, the emphasis was placed on the immediate response to the static loading, rather than the dynamic response, in order to negate thermal effects, which can mask the early signs of damage. The study is focused on the collection of data over a short duration in relatively consistent environmental conditions and controlled loading conditions. The following sections detail the methodology for the collection and analysis of the displacement data, followed by the laboratory testing arrangements. The results captured during the laboratory test program are then analysed using two proposed methods for damage detection, and the findings are presented along with conclusions and recommendations for further work.

## 3. Materials and Methods

### 3.1. Details of Algorithm for Displacement Calculation

The algorithm used to calculate the displacement of the test structures is composed of three elements. First, a scale factor calculation is performed using the dimension correspondences method. This method involves selecting a known physical dimension in the view frame of the camera and measuring it in pixels. A simple conversion is then performed to convert any measurements from pixels to engineering units. This method was used in several studies, and was applied in laboratory and field trials [59,60]. The next step is feature extraction, in which high-contrast keypoints, known as ‘features’, are detected in the image of the structure. The feature extraction was performed using the Speeded Up Robust Features (SURF) [61] algorithm. SURF uses integral images to create a low computation cost scale-space. The scale-space is calculated with the formula:

(1) $$L\left(x,y,\sigma \right)=\;G\left(x,y,\sigma \right)*\left(\;I\left(x,y\right)\right)$$

in which * is the convolution operator, *I(x,y)* is the input image, σ is a scale parameter, and $G\left(x,y,\sigma \right)$ is the Gaussian blur operator, which can be expressed as:

(2) $$G\left(x,y,\sigma \right)=\;\frac{1}{2\pi \sigma^{2}}e^{-\left(x^{2}+y^{2}\right)/2\sigma^{2}}\;\;\;\;\;\;\;\;\;\;$$

**Figure 1:**
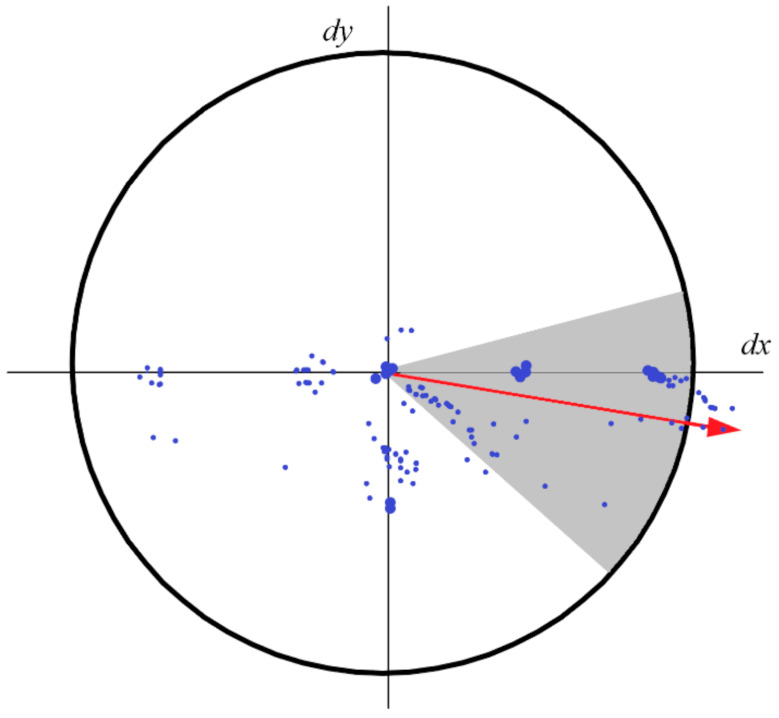
Sliding window used in the SURF algorithm to determine the keypoint orientation.

The original image is then resized to half size, and the Laplacian of Gaussians between the scaled and original image is found. SURF applies a box filter to the integral images in order to approximate this calculation. The determinant of the Hessian matrix *H* for each keypoint is used to detect blob structures, which can be then converted into feature descriptors. For a point x = (x; y) in an image Im, *H*(x; σ) is given by:

(3) $$H\left(x,\sigma \right)=\;\left[\begin{array}{c} \tau_{xx}\left(x,\sigma \right)\;\tau_{xy}\left(x,\sigma \right)\\ \tau_{yx}\left(x,\sigma \right)\;\tau_{yy}\left(x,\sigma \right) \end{array}\right]\;\;\;\;\;\;\;\;\;\;\;$$

in which $\tau_{xx}\left(x,\sigma \right)$ is the convolution of the Gaussian second order derivative $\frac{\partial^{2}}{\partial x^{2}}g\left(\sigma \right)$ with the image *Im* in point *x;* this also applies to $\tau_{xy}\left(x,\sigma \right)$ and $\tau_{yy}\left(x,\sigma \right).$ The box filter of size 9 × 9 approximates a Gaussian with *σ* = 1.2, and represents the lowest level (highest spatial resolution) for blob-response maps. The use of box filters and integral images means that SURF can apply filters of varying size to the image efficiently. Non-maximum suppression is applied in order to determine the location and scale of the keypoints, with the scale space interpolation of the maxima of the determinant of the Hessian matrix performed according to the method proposed by [62]. The orientation of the keypoints is assigned by calculating the Haar wavelets in horizontal and vertical directions for a neighbourhood of size 6 around the location of the point, in which s is the scale the feature was detected in. The Haar wavelet of a region around a point is the sum of the pixel intensities around that point paired with the difference between these sums. This is used as a means of speeding up representations of image regions, as direct calculations of each pixel in the region would be computationally expensive. This method was first proposed by [63]. These responses are then weighted by an adequate Gaussian (*σ* = 2 s), with the dominant orientation estimated by calculating the sum of responses within a sliding window of size 60°, as shown in Figure 1.

A region of 20 s is then created and split into 4 × 4 square sub-regions. In these regions, SURF computes the Haar wavelet responses at 5 × 5 evenly-spaced sample points. The responses are weighted with a Gaussian, and the wavelet responses in the horizontal wav*_x_* and vertical wav_y_ directions for each sub-region are summed to form the first entry of the feature descriptor. The information on the polarity of the intensity change is given by extracting the sum of the absolute values of the responses. By concatenating the descriptor vector *v*, the following is true:

(4) $$v=\left(\sum {\mathrm{wav}}_{x},\;\sum {\mathrm{wav}}_{y},\;\sum \left|{\mathrm{wav}}_{x}\right|,\;\sum \left|{\mathrm{wav}}_{y}\right|\;\right)\;$$

From each sub region, a float-vector descriptor of length 64 is formed. This descriptor can then be used to track keypoints throughout a series of images. Once the features have been detected, they are matched in the subsequent frames of the video by use of the Random Sample Consensus (RANSAC) [64] method of matching features. The basic principle of RANSAC involves drawing a random uniform set of points m (the smallest number of matches required) from the dataset. A least squares line is fitted to this random set, and any points outside a distance (dist) are judged to be outliers. If there are enough points inside the line (inliers) then there is a good fit. This process is repeated *N* times, and the best fit from these tests is used with the fitting error applied as the criteria for the decision. *N* is computed by solving the following equation:

(5) $$N=\;\frac{\log\left(1-P\right)}{\log{\left(1-\varepsilon \right)}^{\rho }}$$

in which ε is the probability that a point is an outlier, *P* is the desired probability that we obtain a good fit, and ρ is the number of points in a sample. A homography between the randomly-selected matching features Ἠ is calculated, and the Ἠ with the highest number of inliers is selected as the final Ἠ. The final Ἠ is applied to the dataset of matched points, and the outliers are removed before the transform estimation is performed. This matching process is repeated throughout the video, and the displacement for the structure over time is calculated. The accuracy of this algorithm was verified in laboratory and field trials by the authors in [65]. 

### 3.2. Details of the Camera Hardware

GoPro Hero 4 [66] action cameras were used to capture the vision-based readings in this study. These cameras were chosen as they are portable, resistant to adverse weather effects, have wireless functionality, and offer an inexpensive and high resolution solution for image capture. A standard GoPro lens would have too short a focal length to be viable for bridge monitoring; a modification [67] kit was used to allow the attachment of C- or F-mount lenses to the GoPro cameras. A Computer [68] ½′′ 25–135 mm F1.8C-Mount lens was attached to the GoPros for the laboratory trial detailed below. 

The footage from each GoPro was synchronized through the use of a Syncbac [69], an accessory that can be attached to the extension port of the GoPro to allow for the embedding of timecode metadata into each frame. The analysis of this metadata allows for the synchronization of the recordings obtained by the system using a solution developed by the authors in C++ in Microsoft Visual Studio. The Syncbac sends live timecode data via Radio Frequency (RF), with a range of 30–60 m. The videos for the laboratory trial were captured in 1080 p.

### 3.3. Damage Detection Approach

The environmental effects, camera resolution, field of view, and image processing algorithms predominantly govern the accuracy of vision-based measurements. A complete derivation of the response of the bridge can rarely be obtained from a single camera, even in the case of a short-span bridge. It is highly likely that multiple synchronized cameras are needed, which is often cost prohibitive. Alternatively, the number of cameras can be reduced when a control vehicle crosses the bridge multiple times, in the following arrangement.

- One camera records displacements of the reference target for each crossing.
- The focus or location of the other monitoring cameras are varied under each vehicle pass event (camera roving).

**Figure 2:**
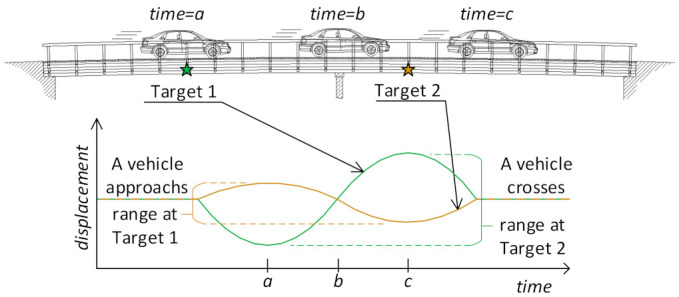
Estimating the displacement range at the target locations.

The structure’s response at a target location over a crossing of a unit load (e.g., a vehicle) is referred to as time histories of target displacement or the unit influence line [70]. Target displacements can be collected at any location on the bridge using vision-based measurement. Capturing the response of all of the targets, when, for example, the reference target reaches the highest displacement, could give erroneous values for some targets that are located far from the load location. The duration of vehicle crossings may vary each time; therefore, any time-dependent response parameters should be excluded when defining a damage-sensitive feature or damage factor. This research proposes to compute the range (i.e., peak-to-peak) of the displacements of each target during vehicle crossings. Figure 2 illustrates the proposed approach on a continuous bridge. Parametric studies were performed by changing the boundary conditions of a scaled bridge model.

Two damage detection methods were applied in this study, and a comparative study was undertaken to establish the advantages and drawbacks of each method, and ultimately to identify the damage conditions by using the displacements obtained from the camera roving runs in all of the experimental cases.

#### 3.3.1. Method 1

In Method 1, the ratio between the difference of the range of target displacement at the baseline ($r_{b}$) and current ($r_{j}$) conditions ($∆r_{j}$) and $r_{b}$ are set as the damage-sensitive feature e (see Equation 6). The feature is expressed as a percentage. When e exceeds the damage threshold, which in this study is set to 5%, the structure is said to be damaged, or the conditions of the structure are altered. Theoretically, if a 5% measurement difference is set as a damage-indicating threshold, even local damages should be detected. The damage is located in whichever e values are the highest.

(6) $$e_{j}=\frac{∆r_{j}}{r_{b}}=\frac{r_{b}-r_{j}}{r_{b}}$$

#### 3.3.2. Method 2

In Method 2, the displacement range of each node in the undamaged case (baseline), and damaged cases can be represented by *r_b_^i^* and *r_d_^i^*, respectively, in which *i* is the node number. The relative displacement ranges of the undamaged case (*R_b_^i^*) and damaged cases (*R_d_^i^*) at Node *i* are defined by

(7) $$R_{b}^{i}=r_{b}^{i}-r_{b}^{1}$$

(8) $$R_{d}^{i}=r_{d}^{i}-r_{d}^{1}$$

The damage factor (DF) is defined as

(9) $$DF^{i}=\frac{R_{d}^{i}-R_{b}^{i}}{R_{b}^{i}}$$

For the identification of the variance in the damage factor at each node in every damage case, the damage condition can be detected. The DF is based on the assumption that the boundary condition change would restrict/increase the displacement of the nodes close to the changed boundary sections. An identified shift in the displacement measurement obtained from the girders acts as an indicator of damage status, and highlights the damage location. 

### 3.4. Laboratory Setup

**Figure 3:**
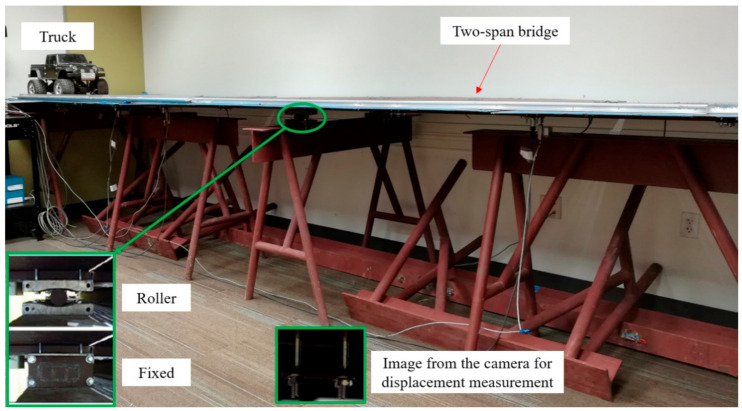
Laboratory setup: a two-span bridge in a laboratory at the University of Central Florida (UCF).

**Figure 4:**
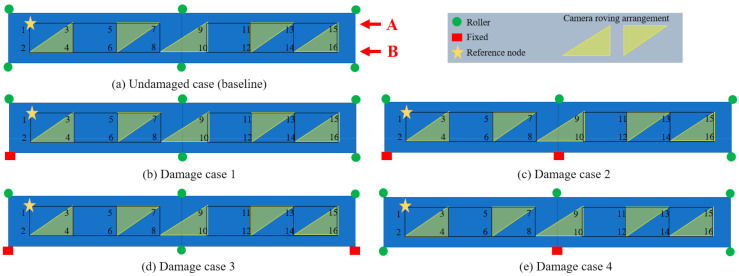
Experimental cases and configurations.

A laboratory model of a two-span bridge was developed in the Experimental Design and Monitoring (EDM) laboratory of Civil Infrastructure Technologies for Resilience and Safety (CITRS) at the University of Central Florida (UCF), as shown in Figure 3. The laboratory bridge model was designed with multiple purposes of investigating structural behaviour under various types of external loads, such as the live loads induced by vehicles crossing the structure, and impact loads induced by the impact hammer [44,71]. The bridge has two 300 cm main continuous spans supported by three steel frame sections, as shown in Figure 3. The bridge deck is a 120 cm wide and 600 cm long of steel plate construction with the thickness of 3.18 mm. The steel deck is supported by two rectangular hollow section (25 × 25 × 3.2 mm) steel girders, with a space of 0.61 m. The girders are denoted as A and B, as illustrated in Figure 4. Connection sets with four M6 bolts and 3.18-mm-thick plates are used to connect the girders and the deck. In this study, a small-scale toy truck with a constant weight was employed as the moving load on the bridge.

The supports of the bridge were varied during the experiment in order to simulate the change of the boundary condition, replicating common real-life bridge conditions. Fixity is considered a type of damage representing the scenario in which falling debris causes the bearing to stick, or in which the roller bearing malfunctions, cannot roll, and then becomes fixed. In addition, adding fixity increases the stiffness of the whole structure, which leads to less displacement.

Four damage cases were designated by changing the supports of Girder B, as shown in Figure 4. Two lanes were predefined on the deck: one was close to Girder A (lane 1) and the other was close to Girder B (lane 2). The truck ran on lane 2, travelling longitudinally from left to right to simulate the moving load. Four cameras were employed to measure the displacements of the predefined measurement nodes on the girders, from 1 to 16, as shown in Figure 4. Each camera was set up using a fixed tripod to measure one node in each run; as such, the measurement of all of the nodes cannot be captured in one run. The orientation of the camera with respect to features to be tracked is important; for this study, the monitoring angle between the cameras and the features was measured using a laser rangefinder, and this angle was accounted for by translating the calculated displacements based on the cosine of the monitoring angle. In the study, the cameras were roved to accommodate all of the measurement nodes. As shown in Figure 4 and Table 1, Node 1 was set as the reference node (Ref. Node), and it is measured in each run. The other cameras were roved in every run; five runs were required to capture all of the measurement nodes. It should be mentioned that such an approach is far more feasible in real life, with four cameras and a test truck with a reasonable number of runs, than with the custom instrumentation and testing.

## 4. Results

The experimental results are presented in this section. Firstly, the measurement pre-processing and the evaluation of the measurement accuracy are presented. Then, the two damage detection methods are applied to the measurement sets.

### 4.1. Measurement Pre-Processing

**Figure 5:**
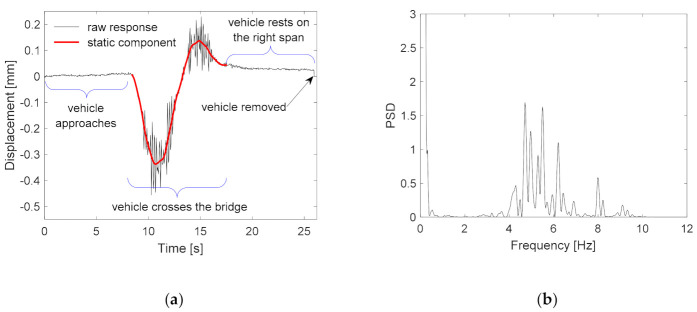
Node 1’s displacement time history (**a**) and its PSD plot (**b**).

The vertical displacements of the Ref Node for the first crossing at the ‘no damage’ condition are shown in Figure 5a. The response pattern is similar to other nodes located on the left span. The vertical displacement histories of the nodes on the right span are opposite the ones on the left support. When the vehicle is on the left span, the right span lifts up, and vice versa. The raw response measurements contain both a static component (the bridge deflection) and a dynamic component (the bridge vibration induced by the vehicle). The static response can be isolated by processing the raw response with an adequate high-pass filter. The conversion of the response measurement from the time domain to the frequency domain reveals the fundamental frequencies. The power spectrum density (PSD) plot is used to set a suitable high-pass filter. The lowest frequency component, which is 0.098 Hz, represents the duration of the vehicle crossing. The crossing lasts approximately 10 s. The frequency range of the dynamic response is above 4 Hz (see Figure 5b). Thus, the high pass frequency is set to 1 Hz. The resulting signal is subtracted from the raw measurements, leaving only the component of the static response. 

**Figure 6:**
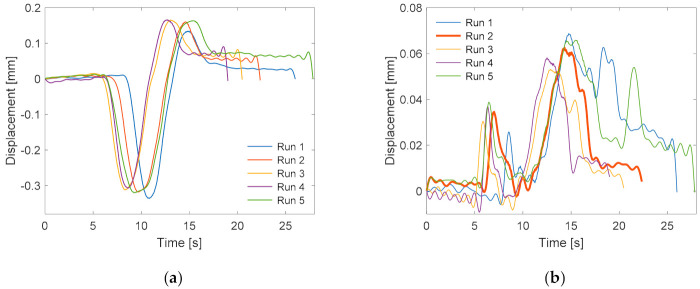
Vertical (**a**) and horizontal (**b**) displacements of the reference node for all of the runs.

The reliability of the computed displacements is scrutinized using the displacements of the reference node for all five runs. The static vertical and horizontal displacements of the reference node are shown in Figure 6**.** The vertical displacements are much more accurate than the horizontal displacements. The statistic showing the mean (μ), standard deviation (σ) and error (σ/μ) of the vertical and horizontal displacement ranges for all of the test cases are given in Table 2. The average horizontal σ ($\sigma_{x}$) for all of the runs is smaller than the vertical σ ($\sigma_{y}$). However, the error is larger for the horizontal displacements. This can be attributed to small movements of the nodes along the horizontal axis, and the achievable resolution with the vision measurement. The table suggests that the largest vertical displacements are observed when no or one support is fixed. The smallest vertical displacements are measured when the support on the left (close to the reference node) and middle of the bridge are fixed. 

Figure 6a shows that all of the runs did not start at the same time, and that they also vary slightly in their duration. For these reasons, selecting the range of vertical displacements of each node as the damage sensitive feature (e) or damage factor (DF) is the best choice. 

### 4.2. Result Analysis by Method 1

**Figure 7:**
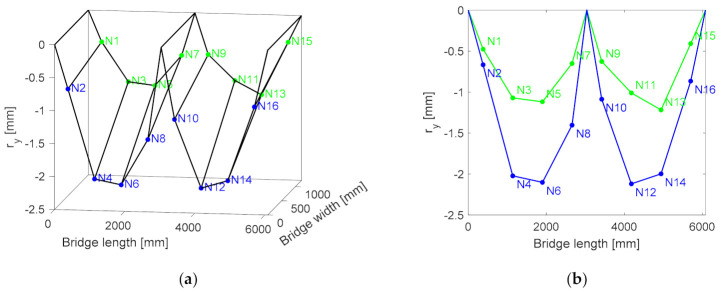
D (**a**) and 2D (**b**) plots of the r values for all of the nodes for the baseline conditions.

For the reference node, $\mu_{y}$ is set as the vertical range ($r_{y}$). Figure 7a shows a three dimensional (3D) plot of the $r_{y}$ values for each node. From here onwards, $r_{y}$ is made negative for visualization purposes. The plot shows that the $r_{y}$ values for the nodes located on Girder B (i.e., Ni, i = 2,4, …, 16) are larger than those for the nodes located on Girder A (i.e., Ni, i = 1, 3, …, 15). This is more apparent in Figure 7b, which is a two dimensional plot of $r_{y}$ along the length of the girder. The bridge response is in accordance to the load application path.

**Figure 8:**
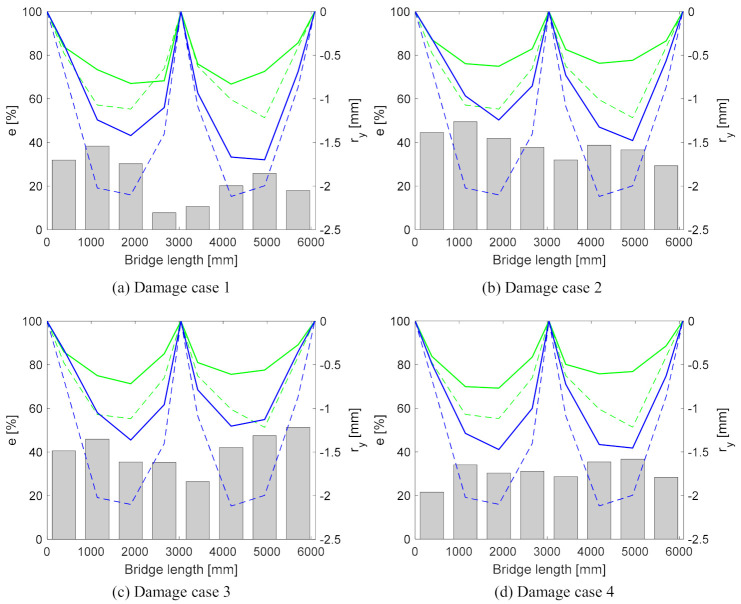
Damage feature (e) (bars) and vertical displacement range ($r_{v}$) expressed as a negative value. The thick green (Girder A) and blue (Girder B) lines are the $r_{v}$ values at the damage cases, with dashed lines at the baseline.

The bridge response at the four damage cases is compared to its baseline (or previous inspection/monitoring) response. The damage-sensitive feature (e) is calculated using Equation (10), which is derived from Equation (6).

(10) $$e=\frac{r_{y,b}-r_{y,d}}{r_{y,b}}\;$$

in which $r_{y,b}$ and $r_{y,d}$ are the sum of the vertical range values for two nodes located at the same distance from the support for the baseline (b) and damage (d) cases, respectively. The bridge response in damage cases is significantly different from its baseline. The e and $r_{y}$ values for all of the damage cases are plotted along the length of the bridge in Figure 8. Although the e values for all of the pairs of nodes along the length of the bridge exceed the 5% damage threshold, very little or no indication of the damage location is discernible. The measurement error was 2%, but it was noted that this was in a laboratory environment with limited change in the environmental conditions. In order to reduce the possibility of false alarms, the damage threshold was set to 5%; the authors do recognise that, in the field, there will be environmental changes. This method is not sensitive to operational variability, as a stabilisation algorithm is applied prior to the processing of the images. The damage threshold can be adjusted according to measurement errors and/or noise for a specific structure by filtering techniques [72]. In field applications, a sensitivity analysis may be required in order to establish the effect of environmental conditions such as temperature, although this will have a limited impact in the change in deflections in the time of a truck passing, which is the premise used in this research.

Only with a prior knowing of damage locations can some conclusions be drawn:

(a) in Damage Case 1, the higher e values are closer to the left support, indicating that left support might be damaged.
(b) in Damage Case 2, all of the e values are higher than in Damage Case 1. The largest amount of high e values are at the left span of the bridge. This could indicate that the bearings at the left support and in the centre are locking.
(c) in Damage Case 3, the smallest e values are at the centre, suggesting that the bearings on the left and right supports are damaged.
(d) In Damage Case 4, the smallest e values are at left and right supports, hinting that the bearing at the centre of the bridge is damaged.

The sum of the e values for Damage Cases 1, 2, 3 and 4 are 182%, 310%, 324% and 246%, respectively. The two largest values, 310% and 324%, are for Damage Cases 2 and 3, when two bearings are fixed.

**Figure 9:**
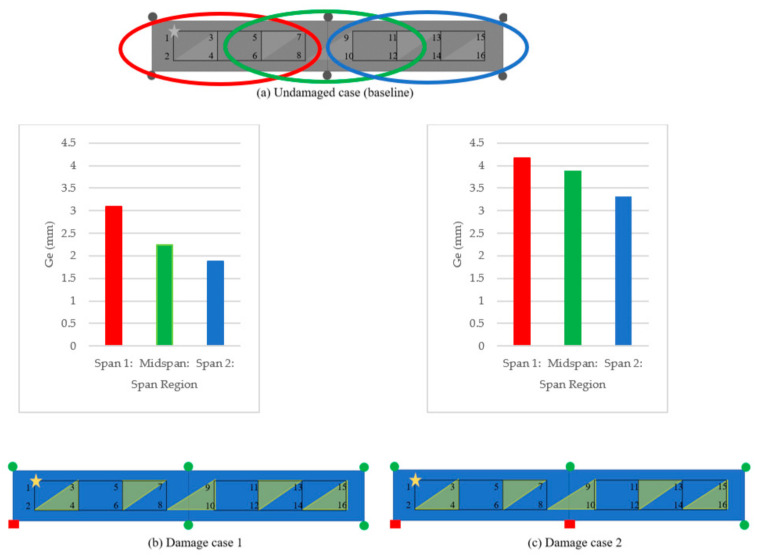

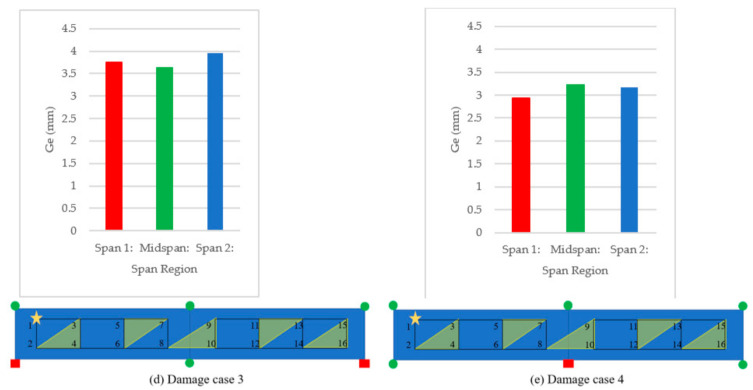
Group damage feature (Ge) for the span regions (bars) at each damage case.

If the absolute difference between $r_{yb}$ and $r_{yd}$ is grouped into zones—as highlighted in Figure 9a, identified as Ge—then it becomes possible to localise the damage to a span region. Based on this, it is assumed that such a dramatic change to a fixed support at any location would have an impact on the global behaviour of the structure. As a result, it is possible that the method cannot localise damage to a node because the physical response is not altered at an isolated node, but rather at a region of the overall of the structure, i.e., Span 1, central, or Span 2. 

**Figure 10:**
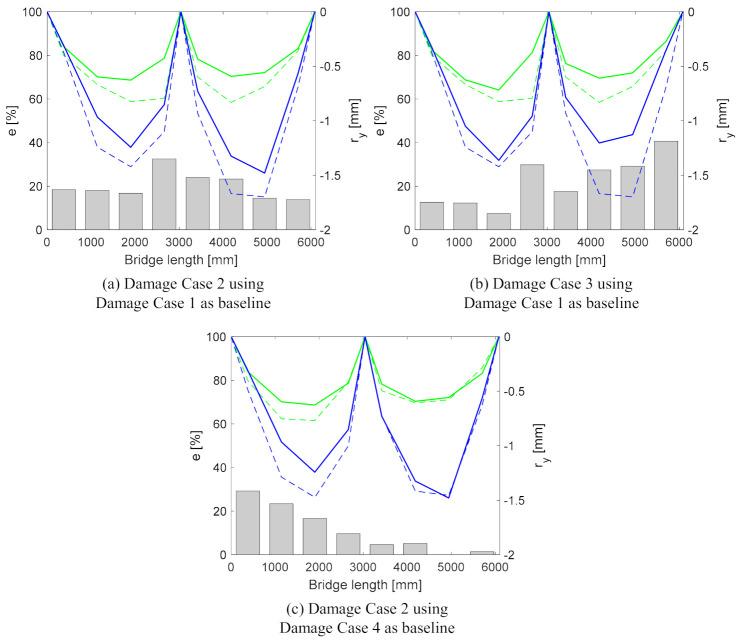
Damage detection using relative changes between different damage cases. The lines and bars describe parameters already introduced in Figure 8.

It is also possible to check the progression of the damage by setting one of the damage cases as the baseline response. The most logical combinations are to set (a) Damage Case 1 as the baseline and analyse Damage Case 2 and 3, and (b) Damage Case 4 as the baseline and analyse Damage Case 3 (see Figure 10) Results show that the best indications of damage locations are for Damage Case 2 (Figure 10c) and Damage Case 3 (Figure 10b).

### 4.3. Result Analysis by Using Method 2 

**Figure 11:**
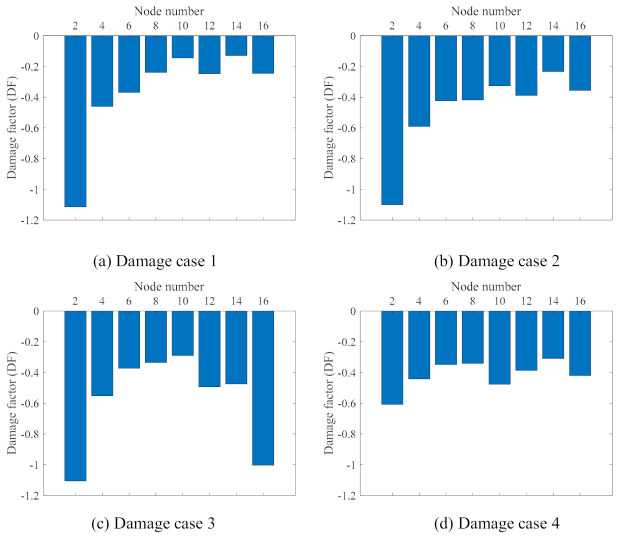
Damage detection result analysis using Method 2.

**Figure 12:**
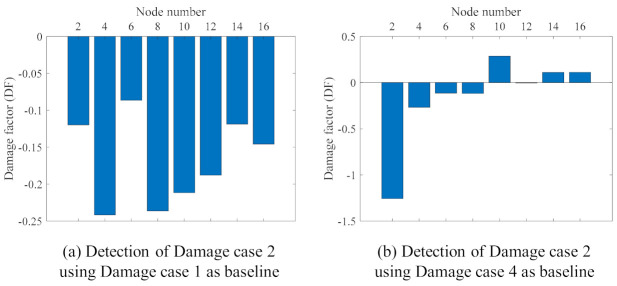
Damage detection using the relative changes between the different damage cases.

In this study, the truck only ran on the lane close to Girder B, and the supports of Girder B were changed to simulate the damages; therefore, here, only the DFs of the nodes (2, 4, 6, …, 16) on Girder B are used to detect the damages. Figure 11 shows the damage detection result using Method 2. The boundary condition being changed from roller to fixed support restricted the displacement of the nodes close to the changed boundary section. As shown in Figure 11a, the DF at Node 2 is clearly identifiable, which can localise the damage to the region close to Node 2. It can be verified by the configuration of Damage case 1 in Figure 4b: the left support of the bridge (close to Node 2) was changed from roller to fixed support. The similar conclusion of the detection of Damage case 3 can also be drawn from Figure 11c and Figure 4d. From Figure 11b, the boundary condition change of the left support—as shown in Figure 4c—can be easily detected. However, the boundary condition change of the middle support in Figure 4c is very hard to recognize. The same is true for Damage case 4, in which only the boundary condition of the middle support is changed, as shown in Figure 4e; it is hard to recognize this damage from Figure 11d. In another view, from Damage case 1 to Damage Case 2, only the boundary condition of the middle support was changed. By setting Damage 1 as the baseline, the DF of Damage case 2 can be calculated, and is shown in Figure 12a. It can be seen that the DF at Nodes 8 and 10—which are close to the middle support—are quite large, and this phenomenon might give some guidance for the detection of the boundary condition change of the middle support. This case results in a low confidence in the damage identification, since the detection DF of Node 4 is of a similar magnitude to those at Node 8 and Node 10. Figure 12b shows the detection of Damage case 2 using Damage case 4 as a baseline. From Damage case 4 to Damage case 2, only the boundary condition of the left support changed from roller to fixed support. The DF in Figure 12b indicated the successful damage identification and localization in this case. 

Overall, by combining Figure 11 and Figure 12, it can be seen that the proposed method, Method 2, can successfully detect and localize the damage of the cases when the boundary condition changes are in the side support, e.g., Damage case 1, Damage Case 3, and the change of the left in Damage Case 4. However, for the damage cases of the boundary condition change at the middle support, Method 2 did not provide adequate confidence in localizing the damages.

## 5. Discussion and Conclusions

The aim of this study was to explore a practical displacement measurement and damage identification method from a limited number of roving vision based sensors, so that engineers can be provided with sufficient warning of bridge damage, which would warrant further inspection or more detailed monitoring. One of the advantages of the proposed method is that it is a potential low cost solution for the contactless monitoring of bridges in the field, which can exhibit a response within the measurement capabilities. It is also well-established that vision-based sensing and monitoring have limitations compared to the conventional sensor (e.g., displacement, acceleration)-based monitoring. However, the low cost and practical aspects that make them applicable to large populations of bridges make them desirable for objective and rapid scans to detect problems. It would be easy to collect and analyse data either in an absolute sense (e.g., a displacement profile evaluation using engineering judgement) or in a relative sense (e.g., based on a baseline or previous set of data). Two methods were used to assess the magnitude of the change in global displacement under changing boundary conditions in the laboratory bridge model. From this study, it was established that either approach could detect damage in the simulation model, but the accurate localisation of the damage was less successful. Method 1—in which the range of vertical displacements for each node in the baseline and damage cases were used—provided a clear indication of damage, but without prior knowledge of the damage cases and laboratory set up, the localization of damage was somewhat inconclusive. However, the authors acknowledge that a limitation of the study was the sole adjustment of the boundary conditions, and neglected to induce localised damage along each of the bridge spans. Therefore, the conclusions of the present study may have been limited by the test conditions. Establishing the ability of each of the methods to detect localised damage along the span of the model requires further research, such as analytical modelling to assess the structural behaviour, and will be addressed in subsequent studies. The authors recognize that, in field applications, the current damage threshold may result in false alarms due to varying environmental conditions. As discussed in Section 4.2, it is the change in deflection under loading that was used for this damage detection technique. It is noted that this was an initial exploration in the use of vision-based measurement for damage detection, and the authors hope to trial this method on a bridge pre- and post-strengthening (which is equivalent to reverse damage).

In Method 2, the displacement ranges of all of the nodes are the relative values calculated by subtracting the displacement range of the reference node. Since the truck loads in the experiments and the boundary changes were made on Girder B, only the damage features calculated by using the displacement ranges of the measurement nodes on Girder B are used in Method 2. Method 2 could give a clear indication of the damage location only for the cases in which the damages were on the side supports. For the cases in which the damage was on the middle support, Method 2 could not localize the damage successfully, probably due to the middle support’s change inducing load distribution along the entire structure. Moreover, Method 2 relies on the quality of measurements of the reference node. If the measurements of the reference node are not good, the damage detection of Method 2 might not be accurate. Setting a response range from a damage case as the baseline response and comparing it with another damage cases can also reveal the damage locations. An example is Figure 10c’s e values, which were generated using Method 1, which increase near and at the left support, in which a bearing is damaged. 

The capture of the full response of a two span bridge using the camera-roving technique for damage detection is presented in this paper, and the damage detection methods show promise in terms of delivering a low cost, no-contact bridge monitoring system suitable for medium- to long-span bridges. The advantage of this approach for damage detection is that it does not require physical sensors to be attached to the structure, and that it negates the requirement for complex sensor installations. The results indicate that, in Method 2, the dependency on a single node for the overall accuracy may result in compromised results in the field; as such, a full scale application of the analysis in this paper on a real bridge is recommended for future work.

## Figures and Tables

**Table 1:** Camera roving arrangement.

No. of Run	Ref. Node	Roved Node	Roved Node	Roved Node
1	1	2	3	4
2	1	5	6	7
3	1	8	9	10
4	1	11	12	13
5	1	14	15	16

**Table 2:** Statistics of the reference node displacements.

	$\mu_{y}\;(\mathrm{mm})$	$\sigma_{y}\;(\mathrm{mm})$	$\sigma_{y}/\mu_{y}\;(\%)$	$\mu_{x}\;(\mathrm{mm})$	$\sigma_{x}\;(\mathrm{mm})$	$\sigma_{x}/\mu_{x}\;(\%)$
Baseline	0.476	0.0049	1.0%	0.067	0.0048	7.2%
Damage case 1	0.403	0.0036	0.9%	0.043	0.0038	8.7%
Damage case 2	0.322	0.0068	2.1%	0.063	0.0053	8.5%
Damage case 3	0.352	0.0049	1.4%	0.026	0.0041	15.8%
Damage case 4	0.405	0.0087	2.1%	0.167	0.0044	2.6%

## Data Availability

Not applicable.
